# Photocatalytic Degradation of Deoxynivalenol Using Cerium Doped Titanium Dioxide under Ultraviolet Light Irradiation

**DOI:** 10.3390/toxins13070481

**Published:** 2021-07-11

**Authors:** Pengzhen He, Zhiyong Zhao, Yanglan Tan, Hengchao E, Minghui Zuo, Jianhua Wang, Junhua Yang, Shuxin Cui, Xianli Yang

**Affiliations:** 1 College of Chemistry and Chemical Engineering, Mu Danjiang Normal University, Mu Danjiang 157012, China; hepengzhenadu@163.com (P.H.); minghuizuo@mdjnu.edu.cn (M.Z.); 2 Institute for Agro-food Standards and Testing Technology, Shanghai Academy of Agricultural Sciences, Shanghai 201403, China; zhaozhiyong@saas.sh.cn (Z.Z.); ehengchao@saas.sh.cn (H.E.); wangjianhua@saas.sh.cn (J.W.); yangjunhua@saas.sh.cn (J.Y.); 3 CAS Key Laboratory of Nutrition, Metabolism and Food Safety, Shanghai Institute of Nutrition and Health, Chinese Academy of Sciences, Shanghai 200031, China; yltan@sibs.ac.cn

**Keywords:** deoxynivalenol, photocatalytic degradation, cerium doped titanium dioxide, mechanism, pathway

## Abstract

Deoxynivalenol (DON) is a major mycotoxin with high toxicity that often contaminates grains, foods and feeds. The traditional approaches for DON removal are difficult to meet industry and agriculture demands due to the high stability of the DON molecule. Therefore, there is an urgent need to develop green and effective strategies for DON degradation. In this study, a batch of photocatalytic nanomaterials of cerium (Ce) doped titanium dioxide (TiO_2_) were successfully prepared by sol-gel method. The catalysts were systematically characterized by XRD, HRTEM, FT-IR, UV-Vis and XPS. The catalyst 0.5Ce-TiO_2_ showed superior photocatalytic activity for DON degradation in aqueous solution under ultraviolet light irradiation, better than that of traditional photocatalyst pure TiO_2_, and 96% DON with initial concentration of 5.0 mg/L could be degraded in 4 h. In addition, the two possible degradation intermediate products C_5_H_8_O_3_ and C_17_H_18_O_6_ were identified, the photocatalytic degradation mechanism and degradation pathway were studied. The results indicate that Ce doped TiO_2_ photocatalyst can be used to reduce DON effectively.

## 1. Introduction

Deoxynivalenol (DON), a high-toxicity secondary metabolite produced by *Fusarium graminearum*, is one of the most common mycotoxins in grains [1], foods and feeds [2]. This mycotoxin poses a serious threat to human health and animals [3,4].

Some technologies have been employed to eliminate DON. Physical methods such as washing and grinding can reduce the content of DON in contaminated grains, but DON is transferred to some other by-products. Heat treatment for DON removal is very limited, due to the strong thermal stability of DON molecule [5]. Bretz et al. reported that the DON molecule could be decomposed under alkaline conditions and then neutralized by chemical agents [6]. Biological detoxification methods could efficiently reduce the toxicity of DON under mild conditions [7]. Yin et al. found a strain A16 isolated from wheat fields, and identified as *Devosia sp*., which could survive and degrade DON in several conditions [8]. Although biological methods have good detoxification effects, the disadvantage is relatively long treatment time, which limits its further application. Therefore, developing a safe and efficient strategy for DON degradation is the trend in food and other related industries and agricultures.

In recent years, more and more researchers have paid increasing attention in the field of photocatalytic technology with high efficiency, low energy consumption and mild reaction conditions for DON degradation [9]. Oxide semiconductor modified doping materials can effectively degrade DON under light conditions, such as ZnO, TiO_2_, etc. Wang et al. prepared photocatalytic materials dendritic-like α-Fe_2_O_3_, which could reduce 90.3% DON with initial concentration of 4.0 μg/mL in an aqueous solution [10]. Bai et al. found the photocatalyst graphene/ZnO hybrids GZ0.3 prepared by a simple one-step hydrothermal method showed good ability to degrade DON in an aqueous suspension under ultraviolet light (UV) light irradiation. Zhou et al. reported a nanoparticles photocatalyst Tm@TiO_2_ composite (UCNP@TiO_2_), which was employed to reduce DON under the simulated sunlight [11]. Additionally, Wu et al. utilized upconversion nanoparticles@TiO_2_ composites UCNP@TiO_2_ to degrade DON in wheat.

Compared to other oxide semiconductor photocatalysts, TiO_2_ as a non-toxic and environmentally friendly nanomaterial with good catalytic degradation of harmful substances has received extensive attention [12]. However, the wide band gap energy of TiO_2_ with about 3.2 eV reduces the efficiency of light utilization and weakens its photocatalytic degradation ability [13]. Metal doping is one of the effective control strategies to enhance the photocatalytic activity by reducing the band gap energy of TiO_2_ [14], which also could inhibit rapid recombination of photogenerated electron-hole pairs and broaden light absorption range redshift due to doping ions in TiO_2_, making it possible to form complexes with Lewis bases such as organic acids or alcohols [15]. Cerium (Ce), as a rare earth element, is relatively nontoxic and cheaper than other rare earth metals. It was doped with TiO_2_ to form a new composite catalysts and can effectively reduce the band gap energy of materials and have better catalytic degradation performance [16].

In this study, we successfully synthesized photocatalytic nanomaterials Ce doped TiO_2_ by sol-gel method. The obtained 0.5Ce-TiO_2_ showed superior photocatalytic activity for DON removal under ultraviolet light irradiation (λ = 254 nm) in an aqueous solution compared with traditional photocatalyst pure TiO_2_. In addition, the possible degradation intermediate products were identified, and the photocatalytic degradation mechanism and degradation pathway were studied.

## 2. Results and Discussion

### 2.1. Structural Characterizations

#### 2.1.1. Morphology Analyses and Crystal Phase

HRTEM Images and XRD Analysis

**Figure 1:**
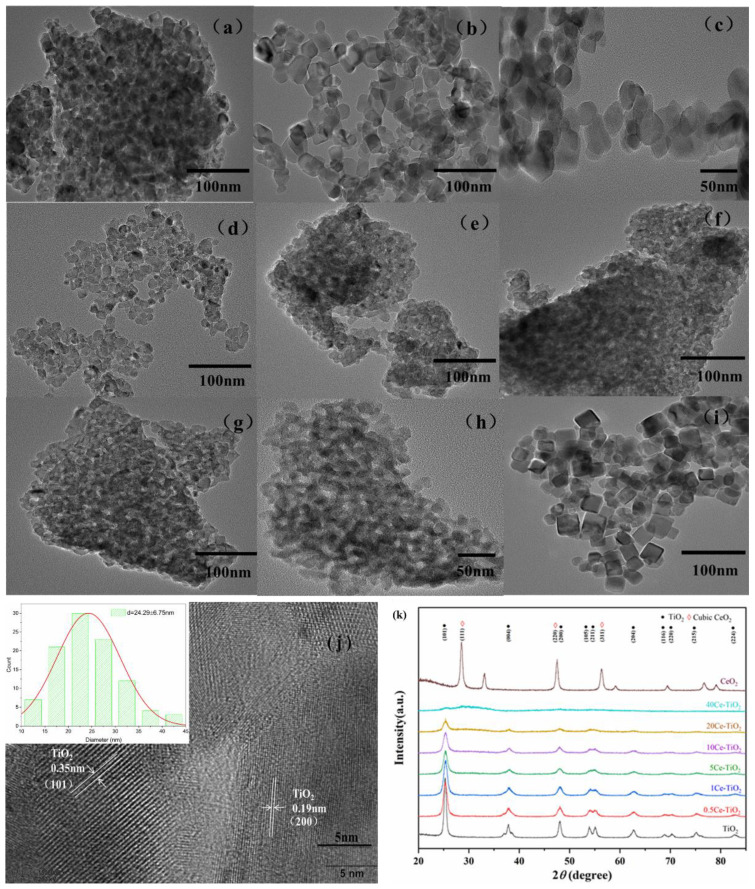
HRTEM images of catalyst prepared by sol-gel method: (**a**) TiO_2_, (**b,c,j**) 0.5Ce-TiO_2_, (**d**) 1Ce-TiO_2_, (**e**) 5Ce-TiO_2_, (**f**) 10Ce-TiO_2_, (**g**) 20Ce-TiO_2_, (**h**) 40Ce-TiO_2_, (**i**) CeO_2_ and (**k**) XRD patterns of as-prepared nanocomposites Ce doped TiO_2_, TiO_2_ and CeO_2_.

In order to study the microstructure and crystal phase composition of Ce doped TiO_2_ catalytic materials, High Resolution Transmission Electron Microscope (HRTEM) were used to characterize the prepared catalytic materials. X-ray diffraction (XRD) was conducted to determine the phases and crystal structures of pure TiO_2_, xCe-TiO_2_(x = 0.5, 1, 5, 10, 20, 40) and CeO_2_. The results are shown in Figure 1.

Figure 1a shows the morphology of the pristine TiO_2_ particles with tiny spherical shapes. Ce doped TiO_2_ shapes appearance becomes uniform and regular with doping Ce, shown in Figure 1b–h. Figure 1j displays the clear lattice fringes of 0.5Ce-TiO_2_ with the particle size of approximately 24 nm. The interplanar distance of approximately 0.35 nm corresponds to the (101) crystal plane of anatase TiO_2_, and the lattice fringe spacing is about 0.19 nm, corresponding to the (220) crystal plane of anatase TiO_2_. Then, the particle size of TiO_2_ doped with 0.5% Ce is about 24 nm, as seen in Figure 1i. Figure 1i shows the morphology of the pristine CeO_2_ particles.

Figure 1i shows that the distinct diffraction peaks at 25.4, 37.9, 48.5, 55.1, 62.8, 68.8, 75 and 82.5° correspond to the (101), (004), (200), (211), (204), (116), (215) and (224) crystal planes of anatase TiO_2_, while the diffraction peaks at 47.4 respond to the (220) planes of the cubic CeO_2_. For the different Ce doping, the XRD patterns demonstrate a similar pattern dominated by anatase, and no other phases can be observed, which is consistent with the HRTEM images. The results are different from previous reports, which showed the formation of the cerium titanate Ce_2_Ti_2_O_7_ phase with Ce:Ti = 50% in molar ratio [17]. Most of the Ce ions cannot enter the TiO_2_ lattice because the radius of Ce^4+^ is larger than that of Ti^4^, and Ce dopant have been well dispersed on the surface of TiO_2_ as the form of cerium oxide [18], which may be the reason why CeO_2_ phases cannot be observed. In addition, the Ce doping expands the TiO_2_ lattice, causing a large lattice distortion and strain energy. In order to compensate the lattice stress, the oxygen atoms on the TiO_2_ surface escape the lattice to form oxygen vacancy, playing a role of trapping holes. The effect may contribute to reduce the probability of recombination of holes and electrons in TiO_2_ and to increase the photocatalytic activity. The main peak (101) broadens with the increase of Ce content, and even disappears, suggests Ce doping inhibits the crystal growth and decreases the crystallinity of the synthesized materials.

XPS Analysis

**Figure 2:**
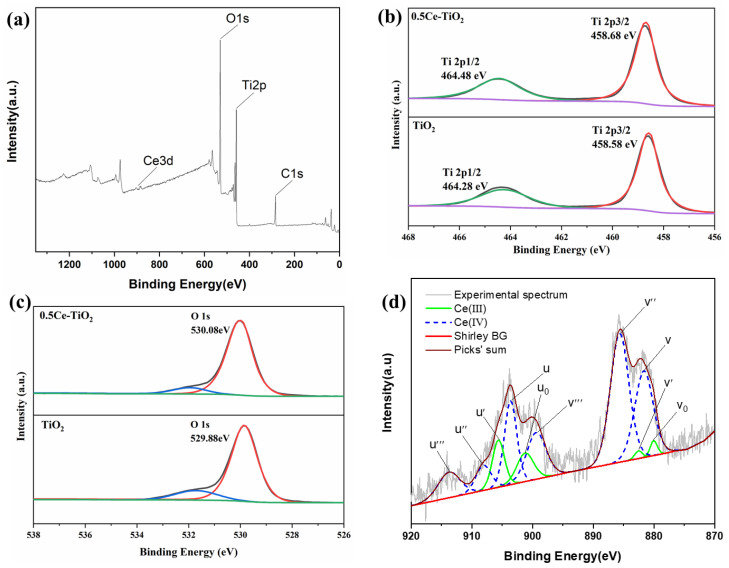
XPS spectra of 0.5Ce-TiO_2_ samples: (**a**) the wide scan spectra, (**b**) Ti 2p, (**c**) O 1s and (**d**) Ce 3d.

The high resolution XPS spectra were performed to analyze and determine the chemical state of the synthesized Ce-doped TiO_2_ photocatalyst. All the obtained spectra were calibrated to the C 1s electron peak at 284.6 eV. The results are shown in Figure 2.

Figure 2a demonstrates the full range survey XPS spectrum of 0.5Ce-TiO_2_ sample. It contains O, Ti and C elements, with sharp photoelectron peaks appearing at binding energies of 531 (O 1s), 458 (Ti 2p) and 285 eV (C 1s). O and Ti are derived from the synthesized photocatalyst, while C 1s is derived from the anhydrous ethanol solvent added during the synthesis process, which fails to volatilize completely during drying and calcination and remains on the surface of the sample. However, it also shows that since there is low doping Ce content, the characterized peak of the Ce element is very weak.

Figure 2b shows the high resolution XPS spectra of Ti 2p for the synthesized sample 0.5Ce-TiO_2_ and pure TiO_2_. The TiO_2_ exhibits two peaks at binding energies 458.58 eV (Ti 2p3/2) and 464.28 eV (Ti 2p1/2), confirming Ti mainly in the Ti^4+^ chemical state [19]. Compared with pure TiO_2_, the two binding energies of Ti 2p after doping Ce show a slight shift for both the two spin orbitals Ti 2p3/2 (458.68 eV) and Ti 2p1/2 (464.48 eV), which indicates a strong interaction of Ti and Ce species [20]. The results suggest the changes in the Ti oxidation states (from Ti^4+^ to Ti^3+^) and confirm that the Ce element is successfully doped into the TiO_2_ structure. Figure 2c shows the O 1s high resolution spectra. The characteristic peak at 529.88 eV in pure TiO_2_ is assigned to the crystal lattice oxygen O^2−^. The main O 1s peak position for 0.5Ce-TiO_2_ is slightly shifted to lower binding energy around 530.08 eV. The reason for peak shifts in O 1s and Ti 2p can be explained by the fact that transferring electrons from O 1s and Ti 2p orbitals to Ce 4f orbitals causes change in the charge densities of the O and Ti atoms [21].

Figure 2d shows the high resolution XPS spectrum of Ce 3d for the synthesized sample 0.5Ce-TiO_2_. The spin orbitals coupling states of 3d5/2 and 3d3/2 are labelled with v and u, respectively. The XPS spectrum of the Ce 3d is relatively complex, mainly due to the hybridization of O 2p and Ce 4f orbital electrons and the partial occupation of the 4f [21]. Hence, the spectrum is categorized into ten constituents. The binding energies of Ce 3d5/2 at 880.06, 881.67, 882.58, 885.66 and 899.39 eV are labelled with v_0_, v, v′, v″ and v″′, while Ce 3d3/2 at 901.22, 903.62, 908.08, 904.12 and 913.74 eV are labelled with u_0_, u, u′, u″ and u″′ [22]. The peaks at v_0_, v′, u_0_ and u′ are characteristic binding energies of Ce^3+^ configurations between the O 2p level and Ce 4f level. v′/u′ is related to the Ce(3d^9^4f^2^) (O 2p^5^) final state, and v_0_/u_0_ is assigned to the Ce(3d^9^4f^1^) (O 2p^6^) final state [23]. The peaks at v, v″, v″′, u, u″ and u″′ are attributed to Ce^4+^. v″′/u″′ is related to the primary photoemission from Ce(3d^9^4f^0^) (O 2p^6^) final state, and v/u is related to the Ce(3d^9^4f^1^) (O 2p^5^) final state [24]. The v″/u″ is from the transfer of two electrons from the O 2p orbital to an empty Ce 4f orbital with the Ce(3d^9^4f^1^) (O 2p^5^) final state [25]. It is deduced that the surface of 0.5Ce-TiO_2_ is not fully oxidized due to the presence of Ce^4+^/Ce^3+^, and the Ce–O–Ti bond may be formed at the interstitial sites or interfaces between CeO_2_ and Ce_2_O_3_, though their contents are too small to be detected by XRD [25].

#### 2.1.2. FT-IR and UV-Vis DRS Analysis

**Figure 3:**
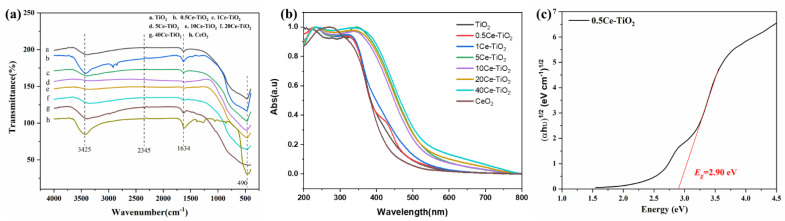
(**a**) FT-IR spectra of TiO_2_, CeO_2_ and Ce-TiO_2_ samples. (**b**) UV-Vis absorption spectra of TiO_2_, CeO_2_ and Ce-TiO_2_ with different Ce ion doping concentration. (**c**) The corresponding band gaps of 0.5Ce-TiO_2_.

Functional groups on the surface of the TiO_2_, CeO_2_ and Ce-TiO_2_ catalyst were analyzed by Fourier Transform Infrared Spectrometer (FT-IR). The UV-vis diffuse reflectance spectra of the materials in the range of 200–800 nm were recorded. The results are shown in Figure 3.

We can see from Figure 3a that the three main characteristic absorption peaks of TiO_2_ are at 490, 1634 and 3425 cm^−1^, respectively, and the three characteristic absorption peaks correspond to the stretching vibration of the Ti-O-Ti bond in the TiO_2_, and the bending vibration and stretching vibration of the O-H bond in the water molecules present on the surface [26,27,28,29]. Comparing the infrared absorption curves of TiO_2_ doped with Ce, the TiO_2_ characteristic absorption peaks in the composites doped with Ce are all present, and the absorption strength of the peaks at 3425 cm^−1^ is enhanced. The results are consistent with the previous reports [29]. Moreover, in the infrared absorption curve of the Ce-TiO_2_, the characteristic absorption peak corresponding to the TiO_2_ at 490 cm^−1^ is shifted, and the position of the peak appears at about 513 cm^−1^. This may be because Ce enters the lattice of TiO_2_ to form Ti-O-Ce bonds, causing effect on the stretching vibration of the original Ti-O-Ti bond. From the figure, we can also see that there is an absorption peak around 2345 cm^−1^ corresponding to CO_2_. The characteristic absorption peak indicates that ethanol did not completely volatilize during the synthesis of the material, and the remaining small amount of inorganic carbon was oxidized to CO_2_ during drying.

According to the spectra, TiO_2_ mainly absorbs ultraviolet (UV) light, and its maximum wavelength is 393 nm. The maximum absorption wavelength of all the Ce-TiO_2_ catalyst samples is shifted to the visible range of 400–600 nm, which expands the range of its absorption spectrum. Moreover, with the increase of Ce content, the red shift of the catalyst samples is more obvious. Among all Ce-TiO_2_ catalyst samples, the red shift of doped metal Ce content is the largest when the content of Ce is 40%. The absorbance of pure TiO_2_ is from the electron transition from O 2p to Ti 3d state. The red shift of Ce-doped TiO_2_ may be caused by the formation a new electronic state, which reduces the distance of charge transfer between 4f electrons of Ce ions and the conduction or valence band in the TiO_2_ bandgap, enhancing the photocatalytic activity [30]. Li et al. have ascribed to Ce^4+^/Ce^3+^ ions as electron scavengers to trap the electrons of TiO_2_ and the Ce 4f level as an interfacial charge transfer and elimination of electron-hole recombination [24].

Comparing with all catalyst samples in the UV region of 200–400 nm, it is found that the doping of Ce can improve the absorbance of TiO_2_, and the absorbance of TiO_2_ increases with the increase of the doping amount of Ce. Band gaps estimated from Tauc transformations of the absorbance spectra are shown in Figure 3c. The band gap (*E_g_*) is calculated mainly by the following formula [31]:

(*αhν*)^1/2^ = K(*hν* − *E_g_*) (1)

where *hν* represents photon energy, *α* represents absorption coefficient, *E_g_* represents band gap energy and K is a constant. It can be seen from Figure 3c that the band gap width of the synthesized 0.5Ce-TiO_2_ sample is 2.9 eV, less than that of TiO_2_ (*E_g_* = 3.2 eV). The results indicate that doping Ce in TiO_2_ significantly reduces the band gap energy and effectively inhibits the recombination of photogenerated electrons-holes.

### 2.2. Photocatalytic Degradation of Deoxynivalenol

#### 2.2.1. Evaluation of the Effectiveness of DON Degradation

**Figure 4:**
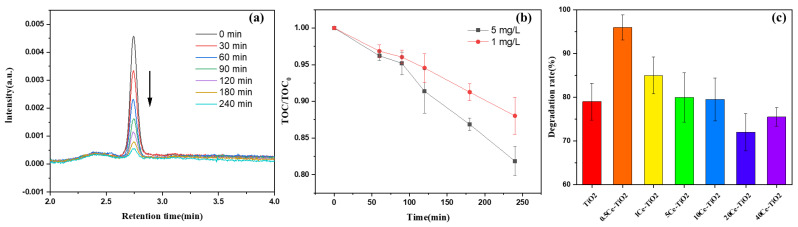
(**a**) HPLC chromatogram of DON photodegradation using 0.5Ce-TiO_2_. (**b**) TOC removal in DON aqueous solution of different initial concentration. (**c**) The degradation rate of different photocatalysts under UV light irradiation for DON removal after 240 min.

The effectiveness of Ce-TiO_2_ photocatalytic degradation DON under UV light (λ = 254 nm) and the total organic carbon (TOC) changing trends during the reaction of photocatalytic degradation DON in aqueous solution using 0.5Ce-TiO_2_ are shown in Figure 4.

The rapid photocatalytic degradation of DON over 0.5Ce-TiO_2_ nanomaterials was clearly seen by using High Performance Liquid Chromatography (HPLC), as shown in Figure 4a. The HPLC chromatograms show decreasing DON peaks at retention time 2.74 min with prolonging irradiation time. The Figure 4c shows the different photocatalytic degradation effects of Ce-TiO_2_ with different Ce doping content and pure TiO_2_. The optimum doping amount of Ce is 0.5% in our study. The degradation rate of the DON aqueous solution at 5 mg/L can reach 96% using 0.5Ce-TiO_2_ under UV light irradiation after 240 min. The photocatalytic degradation effect is higher than 85% under the same conditions. The results indicate that the small CeO_2_ particles produced on the TiO_2_ particles caused by Ce doping play a co-catalytic effect on DON degradation. The removal effects of 5Ce-TiO_2_ and 10Ce-TiO_2_ are equivalent to pure TiO_2_. However, the degradation rate of 10Ce-TiO_2_ and 40Ce-TiO_2_ are lower than that of TiO_2._ The main reason may be that excessive Ce destroy the TiO_2_ lattice structure and decrease the photoactivity of the nanomaterials, although the light absorption range redshifts [32]. The results indicate that 0.5Ce-TiO_2_ has superior photocatalytic activity for DON removal under UV light (λ = 254 nm) irradiation, which is even better than that of the traditional photocatalyst TiO_2_.

In the course of the degradation, the measurement of TOC is an important embodiment of the mineralization reaction and the mineralization degree. DON aqueous solution with initial concentrations of 1 mg/L and 5 mg/L were selected here. It can be seen from Figure 4b that the TOC values are gradually decreasing with prolonging the photocatalytic reaction, which means that the mineralization rate of the DON aqueous solution is increasing with the reaction time increasing. The results indicate that more and more DON molecules are further oxidized to H_2_O and CO_2_ completely. The TOC/TOC_0_ ratio of 5 mg/L is less than that of 1 mg/L resulted from more molecules involved in the degradation reaction in the high concentration of the DON aqueous solution.

#### 2.2.2. Free Radical Trapping Experiments and Photocatalytic Degradation Mechanism

**Figure 5:**
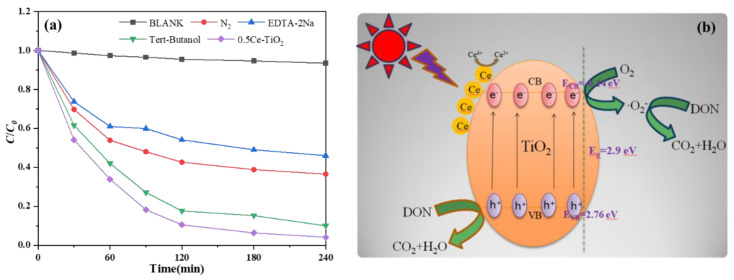
(**a**) The various scavengers’ effects on the photocatalytic degradation of DON using 0.5Ce-TiO_2_. (**b**) Schematic illustration for the charge separation and transfer of Ce-TiO_2_ in the process of DON degradation under UV light irradiation.

To explore the main active substances in the process of DON degradation, the active material capture experiments were carried out. We choose EDTA-2Na as hole (h^+^) scavenger [33], tert-butanol as the hydroxyl radical (•OH) [34] and nitrogen (N_2_) bubbling was used to superoxide radicals (•O_2_^−^) trapping agents. The results are shown in Figure 5a.

From Figure 5a, the photocatalytic activity of 0.5Ce-TiO_2_ decreases largely by the addition of hole scavenger (EDTA-2Na), while no significant decrease was observed by the addition of •OH scavengers, indicating that •OH are not the main oxidative species affecting catalytic degradation. That is, the hole plays a more key role in the photocatalytic degradation reaction than •OH in the UV light irradiation [35]. In addition, the degradation efficiency of DON using 0.5Ce-TiO_2_ is obviously reduced with the anoxic solution, indicating that O_2_ is another more important role in the photodegradation reaction that produces more •O_2_^−^, which is consistent with the previous study [36]. Based on all the results above, we can conclude that the photooxidation mechanism occurring on the surface of 0.5Ce-TiO_2_ may involve in the direct oxidizing reaction of DON with •O_2_^−^ and holes. The photocatalytic degradation mechanism of the 0.5Ce-TiO_2_ sample may involve the oxidation process of holes and •O_2_^−^.

The photocatalytic degradation mechanism for DON removal using Ce-TiO_2_ nanomaterials synthesized in this experiment for DON are shown in Figure 5b. The electrons in the valence band (VB) of the Ce-TiO_2_ sample are excited to the conduction band (CB) under the irradiation of UV light. The number of holes on VB is the same as the number of electrons on CB. Under general conditions, photogenerated electrons-hole carriers are easily inclined to recombine, resulting in only a small part of the electrons involved in the catalytic degradation process [37]. The band position of doped samples is mainly calculated by the following formulas [38]:

*E_VB_* = *χ* − *E^e^* + 0.5*E_g_* (2)

*E_CB_* = *χ* − *E^e^* − 0.5*E_g_* (3)

where *E_VB_*, *E_CB_*, *χ* and the *E_g_* are the VB edge potential, CB edge potential, Sanderson electronegativity and the band gap of the photocatalysts. The value of *χ* for TiO_2_ is 5.81 eV, and *E^e^* represents the free electron energy on the hydrogen scale, with a value of 4.5 eV. According to the above formula, the *E_CB_* of Ce-TiO_2_ is −0.14 eV and the *E_VB_* is 2.76 eV. In this material, Ce dopants into the TiO_2_ lattice introduce new impurity levels (empty Ce 4f) with a smaller band gap close to the Ti 3d conduction band of TiO_2_ [32]. Under light illumination, the distance of the excited charge carrier transfer from Ti 3d of TiO_2_ to Ce 4f level is narrowed [39], which can reduce the charge carrier’s recombination rate [40]. In addition, the photogenerated electrons on the conduction band react with O_2_ to form •O_2_^−^, and holes in VB react with DON to form CO_2_ and H_2_O. The reaction formula is:

*e*^−^ + O_2_ → •O_2_^−^ (4)

*h*^+^ + -OH → •OH (5)

DON + *h*^+^ → CO_2_ + H_2_O (6)

DON + •O_2_^−^ → CO_2_ + H_2_O (7)

#### 2.2.3. Intermediate Products of Don Degradation and Possible Photodegradation Pathway

The intermediate products of DON photocatalytic degradation were analyzed using by Ultra Performance Liquid Chromatography coupled with Quadrupole Time-of-flight Mass Spectrometry (UPLC-QTOF/MS) (ESI^+^ mode).

**Figure 6:**
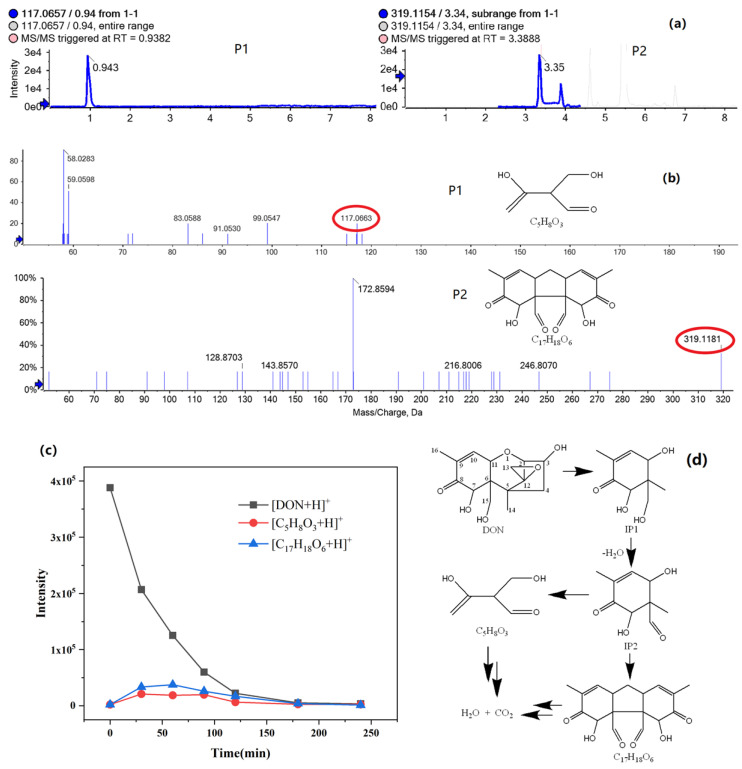
(**a**) Extracted Ion Chromatogram (XIC) of intermediate products P1 and P2. (**b**) MS/MS spectrum of intermediate products P1 and P2. (**c**) The intensity changing trends of the molecular ion peak of [DON+H]^+^, [P1+H]^+^ and [P2+H]^+^ with the reaction time. (**d**) The pathway of DON degradation.

As shown in Figure 6a, Extracted Ion Chromatogram (XIC) shows the two intermediate products’ peaks. The two intermediates chemical structures P1 C_5_H_8_O_3_ (m/z theoretical 117.06, experimental 117.07) and P2 C_17_H_18_O_6_ (m/z theoretical 319.12, experimental 319.12) are identified by comparing with the DON (m/z theoretical 297.00) molecule, as shown Figure 6b. We speculate that P1 and P2 structures are from a possible unstable intermediates (IP1) six-membered ring compound via opening loop with C1-C2 bond fractures and by removing the five-membered ring. The five-membered ring with a 12,13-epoxy group is one of the main toxic functional groups in DON [41]. The reaction can eliminate the toxicity of DON. However, we did not find that the carbonyl-containing intermediate product ion (m/z 303.09) formed by the epoxy ring group destroyed, which is different from the previous study [42] due to the strong oxidizing ability. Then, IP1 dehydrates to form the relatively stable compound IP2. Two possible reaction pathways may exist. One is that IP2 continue to be oxidized to form P1. The other one is that the two IP2 molecules are coupled to form P2. The intensity changing trends of the molecular ion peak of [DON+H]^+^, [P1+H]^+^ and [P2+H]^+^ with the reaction time are shown in Figure 6c. Because TOC content gradually decreases with the reaction time increasing, DON is further oxidized and mineralized to CO_2_ and H_2_O completely. The possible pathway that intermediate products P1 and P2 generated from DON molecular break, continued to react and finally disappeared with the photocatalytic degradation reaction is speculated and shown in Figure 6d.

## 3. Conclusions

In conclusion, TiO_2_ photocatalytic nanomaterials doped with Ce were successfully prepared by the sol-gel method. In these synthesized materials, 0.5Ce-TiO_2_ shows superior photocatalytic activity for DON removal in aqueous solution under UV light irradiation (λ = 254 nm). The free radical trapping experiments indicate that the photogenerated h^+^ and •O_2_^−^ are the two main active substances for DON photocatalytic degradation. The two possible degradation intermediate products C_5_H_8_O_3_ (m/z 117.07) and C_17_H_18_O_6_ (m/z 319.12) were identified, which indicates that the main toxic groups in the DON molecule were destroyed. This work provides an efficient and mild method to reduce DON contamination. In order to further evaluate the feasibility of this method, the study on toxicity of DON degradation products is ongoing.

## 4. Materials and Methods

### 4.1. Materials

Tertbutyl titanate (TBOT), cerium nitrate hexahydrate (Ce(NO_3_)_3_•6H_2_O, 99%), tert-butyl alcohol (TBA), EDTA-2Na and acetic acid were purchased from Shanghai Titan Technology Co., Ltd. Water used in the experiment was ultrapure water prepared from the Milli-Q system (Millipore, Billerica, MA, USA). Acetonitrile and methanol (mass spectrum grade) were purchased from Fisher Chemical.

### 4.2. Synthesis of Ce-TiO_2_, TiO_2_ and CeO_2_

Ce-dropped TiO_2_ (Ce-TiO_2_) based catalysts were prepared using the sol-gel method. In the study, tertbutyl titanate (TBOT) and cerium nitrate hexahydrate (Ce(NO_3_)_3_•6H_2_O) were used as the reaction precursors. The typical synthetic procedure was as follows: first, 15 mL TBOT and little amounts of acetic acid were dissolved in 30 mL absolute ethanol to make the solution A. The solution B was cerium nitrate hexahydrate aqueous solution. Solution B was dropped into solution A slowly with vigorous stirring. The mixed solution continued to stir, and the solution became sol and gel. The gel stood for 12 h, and then was dried at 120 °C in an oven; finally, it was calcined at 550 °C for 3 h. The product was Ce-TiO_2_. Using the same synthesis procedure, the product with different proportion was obtained by only changing the content of cerium nitrate (the Ce contents were varied as Ce: Ti = 0.5, 1, 5, 10, 20 and 40% in molar ratio) and was marked as 0.5Ce-TiO_2_, 1Ce-TiO_2_, 5Ce-TiO_2_, 10Ce-TiO_2_, 20Ce-TiO_2_ and 40Ce-TiO_2_, respectively. In addition, pure TiO_2_ nanomaterials were synthesized without adding cerium nitrate as a control. The CeO_2_ catalyst was also prepared by sol-gel method [43].

### 4.3. Characterization of Catalysts

The crystal structure of the sample was determined by X-ray diffraction (Bruker D8 ADVANCE, Germany), with Cu Kα radiation as the X-ray source, and operated at 40 kV and 40 mA. The 2*θ* scan range was 20–85° with a step size of 0.02°. Morphology and structure of catalysts were observed using high-resolution transmission electron microscopy instruments (JEM-2100F, Japan). X-ray photoelectron spectroscopy (XPS) studies were performed with a Escalab 250Xi spectrometer (ThermoFisher, MA, USA), using a mono-chromatic Al Kα source. The infrared spectra were recorded on the Nicolet iS5 FT-IR Spectrometer (Thermo Scientific, WI, USA). The UV-Vis diffuse reflectance spectra of 200–800 nm were recorded on the Shimadzu UV 3600plus (Shimadzu, Japan).

### 4.4. DON Photocatalytic Tests

Photocatalytic tests were carried out in photochemical reaction apparatus, and the tests were performed using a UV lamp (254 nm). The initial concentration of DON solution was 5 mg/L. Before the light irradiation, 2.5 mg catalyst was added to 20 mL of DON solution and kept in the dark for 30 min to reach the adsorption-desorption equilibrium. After different irradiation times (30 min, 60 min, 90 min, 120 min, 180 min and 240 min), 1 mL of suspension was collected and centrifuged at high speed. The upper liquid was filtered through a 0.22 μm filter membrane and was analyzed. The concentration of DON was determined by Acquity Ultra Performance LC (Waters, Milford, MA, USA) equipped with a Waters Acquity BEH C18 column (1.7 μm, 2.1 × 100 nm) and with an isocratic mobile phase composed of methanol-water (20:80) at a flow rate of 0.25 mL/min. The DON degradation rate was calculated by the following formula.

*η*(%) = (*C*_0_ − *C*_t_)/*C*_0_ × 100 (8)

where *C*_0_ represents the initial concentration of DON, and *C*_t_ represents the concentration of DON after photocatalytic degradation.

Seven DON aqueous solutions of 20 mL with the same initial concentration 1 mg/L and 5 mg/L were, respectively, irradiated at different irradiation times (0 min, 30 min, 60 min, 90 min, 120 min, 180 min and 240 min) and directly submitted to TOC analysis. The TOC content was determined by Total Organic Carbon Analyzer TOC-L CPH Basic System (Shimadzu Co. Ltd., Kyoto, Japan).

To investigate the photocatalytic mechanism of DON degradation, the active species trapping experiment was performed with three different active substance capture agents (N_2_, ethylenediaminetetraacetic acid disodium salt dihydrate (EDTA-2Na) and tert-butyl alcohol). The other conditions remain unchanged in this experiment.

### 4.5. Degraded Intermediate Products Analysis

The identification of the intermediate products of DON was performed by AB SCIEX 5600 Triple TOF mass spectrometer (Foster City, MA, USA). A Waters Acquity BEH C18 column (1.7 μm, 2.1 × 100 mm) was used for chromatographic separation at a flow rate of 0.3 mL/min. The mobile phase consisted of methanol (A) and water (B). A linear gradient elution program was used as follows: 0 –2.0 min, 5% A; 2.0–15.0 min, 5–95% A; 15.0–17.0 min, 95% A; 17.0–17.1 min, 95–5% A; 17.1–20.0 min, 5% A. The mass spectrometer conditions were as follows: ion spray voltage, 5.5 kV; block source temperature, 500 °C; ion source gas 1 pressure, 50 psi; ion source gas 2 pressure, 50 psi; curtain gas pressure, 35 psi. TOF/MS scan was performed in the mass range of m/z 50–400 with the collision energies of 10 and 45 eV. Data processes were performed using PeakView version 2.1 and MasterView version 1.0 (AB Sciex, Framingham, MA, USA).
